# Transfer Learning Models for Detecting Six Categories of Phonocardiogram Recordings

**DOI:** 10.3390/jcdd9030086

**Published:** 2022-03-16

**Authors:** Miao Wang, Binbin Guo, Yating Hu, Zehang Zhao, Chengyu Liu, Hong Tang

**Affiliations:** 1 School of Biomedical Engineering, Dalian University of Technology, Dalian 116024, China; wangmiao9248@mail.dlut.edu.cn (M.W.); guobinbin@mail.dlut.edu.cn (B.G.); yatinghu@mail.dlut.edu.cn (Y.H.); zhaozehang@mail.dlut.edu.cn (Z.Z.); 2 School of Instrument Science and Engineering, Southeast University, Nanjing 214135, China; chengyu@seu.edu.cn

**Keywords:** heart sound signal, continuous wavelet transform, transfer learning, data augmentation, multiple label classification

## Abstract

Background and aims: Auscultation is a cheap and fundamental technique for detecting cardiovascular disease effectively. Doctors’ abilities in auscultation are varied. Sometimes, there may be cases of misdiagnosis, even when auscultation is performed by an experienced doctor. Hence, it is necessary to propose accurate computational tools to assist auscultation, especially in developing countries. Artificial intelligence technology can be an efficient diagnostic tool for detecting cardiovascular disease. This work proposed an automatic multiple classification method for cardiovascular disease detection by heart sound signals. Methods and results: In this work, a 1D heart sound signal is translated into its corresponding 3D spectrogram using continuous wavelet transform (CWT). In total, six classes of heart sound data are used in this experiment. We combine an open database (including five classes of heart sound data: aortic stenosis, mitral regurgitation, mitral stenosis, mitral valve prolapse and normal) with one class (pulmonary hypertension) of heart sound data collected by ourselves to perform the experiment. To make the method robust in a noisy environment, the background deformation technique is used before training. Then, 10 transfer learning networks (GoogleNet, SqueezeNet, DarkNet19, MobileNetv2, Inception-ResNetv2, DenseNet201, Inceptionv3, ResNet101, NasNet-Large, and Xception) are used for comparison. Furthermore, other models (LSTM and CNN) are also compared with our proposed algorithm. The experimental results show that four transfer learning networks (ResNet101, DenseNet201, DarkNet19 and GoogleNet) outperformed their peer models with an accuracy of 0.98 to detect the multiple heart diseases. The performances have been validated both in the original heart sound and the augmented heart sound using 10-fold cross validation. The results of these 10 folds are reported in this research. Conclusions: Our method obtained high classification accuracy even under a noisy background, which suggests that the proposed classification method could be used in auxiliary diagnosis for cardiovascular diseases.

## 1. Introduction

Heart disease morbidity and mortality are increasing year after year. Meanwhile, heart disease has become a serious disease threatening human health. There are various methods for diagnosing cardiovascular diseases [1]. Among them, the most common methods are electrocardiograms (ECG) and phonocardiograms (PCG), which are used for detection of heart diseases. ECG can evaluate the condition of the heart work directly. However, in some cases, the ECG cannot reflect all existing disorders, such as the presence of heart murmurs [2].

In the clinical examination, the doctors first listen to the sounds on the surface of the patients’ chest by the stethoscope. These sounds are called heart sounds (HSs), and the recording of the HSs is called phonocardiogram (PCG). The PCG can reflect the condition of the cardiovascular system comprehensively, which contains pathological or physiological information of the heart. Therefore, PCG has great value in assisting doctors to diagnose or analyze different kinds of heart diseases [3].

As we know, there are four valves (the mitral valve, tricuspid valve, pulmonary valve, aortic valve) in the heart. If there is a problem with these heart valves opening or closing, there will be damage to the heart which may cause heart valve disease. Heart valvular diseases usually involve mitral stenosis (MS), mitral regurgitation (MR), aortic stenosis (AS), and mitral valve prolapse (MVP). These different heart valvular diseases reflect different features on heart sounds.

Mitral stenosis: Mitral stenosis will cause rheumatic heart disease. Diastolic blood flows from the left atrium through the narrow mitral valve to the right ventricle. It will generate low-pitched murmurs. The murmur can be heard best at the apex.

Mitral regurgitation: The murmur of mitral regurgitation is generated as blood regurgitates from the left ventricle to left atrium. The first heart sounds (S1s) are very soft. We can hear a pan-systolic murmur best at the apex of heart. 

Aortic stenosis: The murmur of aortic stenosis is a systolic ejection murmur that peaks early in systole. It is heard best at the second right interspace.

Mitral valve prolapse: If mitral valve prolapse is present, then a mid-systolic click may be heard, followed by a late systolic murmur.

In addition, we also provide the data on patients of pulmonary hypertension (PH).

Pulmonary hypertension: PH is a hemodynamic and pathophysiological condition in which the pulmonary artery pressure rises above a certain threshold. Symptoms of heart sound findings include augmented second heart sound (such as P2 component), tricuspid regurgitant, and the third heart sound (S3) gallop.

However, doctors are not always able to diagnose heart diseases accurately by simply listening or observing a HS record. For this reason, studies on PCG have been increased to make it easier for doctors to make a diagnosis. In recent years, computer-assisted detection technology for the processing and analysis of heart sound signals have made remarkable achievements and aroused great interest [4,5,6,7,8].

Currently, smart detection of PCG technology has not been widely used in real-life clinical diagnosis, and the main method used for detection of heart sounds is still artificial auscultation. Therefore, research and application of computer-aided heart sound detection techniques will greatly facilitate the development in the field of cardiovascular disease diagnosis. From the existing research literature, there were mainly four strides used to detect cardiovascular disease: (1) pre-processing of the heart sound signals, (2) segmentation of the first heart sounds (S1s) and the second heart sounds (S2s) or division of cardiac cycles, (3) extraction of features, and (4) recognition of normal and abnormal HS recordings. In general, manual operation or algorithms extract the key features from PCG signals first. Then, they compare the monitoring sequence of the patients with the tagged database. At last, more intuitive diagnostic results can be obtained automatically.

In early years, many researchers paid close attention to the location of the boundaries of HS components (such as: S1s and S2s) [9,10,11,12,13,14,15]. However, these segmentation methods may be inaccurate with the massive growth of databases today. If the segmentation is inaccurate, then the detection of cardiovascular disease will even be more inaccurate. Therefore, most of the current methods are based on feature extraction to detect heart diseases instead of segmentation of S1s and S2s. In our research, we classify the PCGs without segmentation of HSs.

In the feature-extraction stage, it is worth noting that some of the features of one-dimensional signals are similar in diverse cardiovascular diseases. These similar features may influence the results of the multi-classification. As a consequence, it is particularly important to magnify the variedness in different features of the heart diseases. Many researchers have extracted manual features [16,17,18]. Most of these handcrafted features have physiological explanations, such as the amplitude, time interval, kurtosis, energy ratio, MFCC, and entropy etc. These features have usually been used to conduct binary classification (normal PCG vs. abnormal PCG) by previous researchers. The computation of these manual features is small and simple, but may be not good at multi-classification and new databases. For deep networks with complex and deep structures, the classification effect may be poor. Hence, deep features are needed for multi-classification of heart diseases. Some researchers have used deep-learning models to extract deeper features automatically, such as CNN or other ANN models [19,20,21]. Additionally, their results were better than the results of manual features extraction.

Table 1 shows a detailed comparison with some recent existing excellent work. However, there are some limitations in the field of HSs classification due to the few clinical databases. Additionally, most of the studies have focused on binary classification. At the same time, most of the validation and training was based on one single database (such as PASCAL or Open heart sound database). This is because of the absence of multi-labels heart sound databases and corresponding annotations of the categories of heart sounds from the databases. To solve this problem, we combine the databases from the website [22] with the data collected by ourselves together, which have six categories of heart sound signals in total (normal, mitral stenosis, mitral regurgitation, mitral valve prolapse, aortic stenosis and pulmonary hypertension). Furthermore, in our research, the proposed method is validated based on data augmentation condition. This works very well under the different noise recordings based on the heart sound augmentation method.

This paper is organized as follows: Part 2 introduces the two databases applied in our research. Part 3 describes the detailed method, such as the CWT for the creation of the time–frequency images and the transfer learning models with the augmented databases. Part 4 describes the results of the 10 transfer learning models. At the same time, the transfer learning models have compared the results with other multi-classification results. Part 5 contains the conclusion of this paper’s proposed method.

## 2. Database Details

(Database A) The phonocardiogram database [22] is used as one of our databases. The database includes 1000 audio recordings (the exact number of people is unclear) which are in the format of wav audio. The sampling rate is 8000 Hz. There are five categories of heart sound signals, which are normal (N) and four major valvular heart diseases: mitral stenosis (MS), mitral valve prolapse (MVP), mitral regurgitation (MR), aortic stenosis (AS). Each category has 200 HS recordings (200 audio recordings/per category). The duration of heart sound signals ranges from 1.1556 s to 3.9929 s in the database A. We take the HS signal time length up to 1.1556 s according to the minimum time length of HS signal in database A. The database of the five categories of original heart sounds can be obtained at: https://github.com/yaseen21khan/Classification-of-Heart-Sound-Signal-Using-Multiple-Features-/blob/master/README.md (accessed on 10 September 20212).

(Database B) The second database was collected at the Second Hospital of Dalian Medical University. All the subjects were informed to the study and signed the study participation consent. The database B contains 74 PH subjects, including 102 recordings in total. The sampling rate is 2000 Hz. We select two non-overlapping segments according to the time length of 1.1556 s from each original recording randomly in database B. Therefore, there are 204 recordings after we re-split the recordings. To be consistent with the numbers in database A, we select the first 200 heart sound recordings as database B.

**Figure 1:**
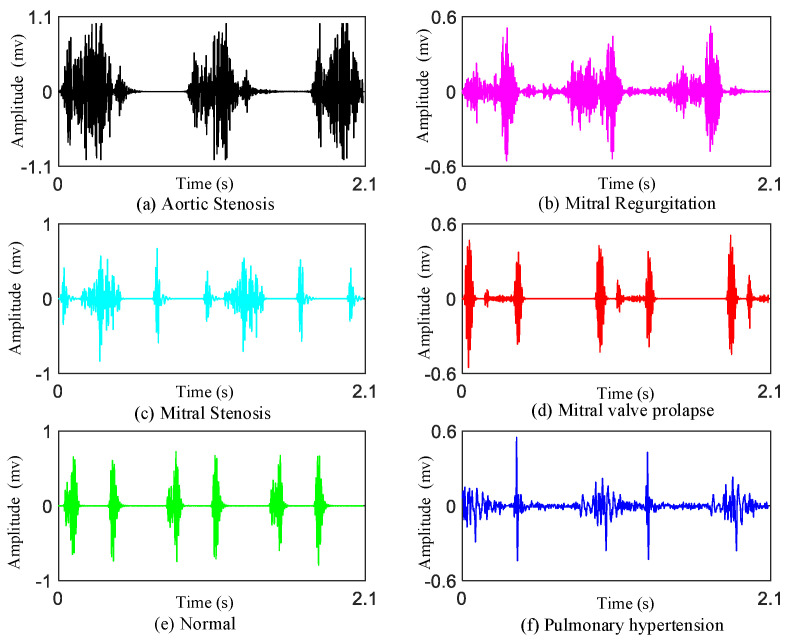
Typical examples of the PCG signals. (**a**) One example of AS; (**b**) One example of MR; (**c**) One example of MS; (**d**) One example of MVP; (**e**) One example of N; (**f**) One example of PH.

The details of the two given databases are described in Table 2. Additionally, typical examples of the PCG signals of the represented classes are shown in Figure 1. The PH database can be obtained at: https://github.com/wangmiao1992/pulmonary-hypertension-database/tree/main (accessed on 12 January 2022).

## 3. Methodology

**Figure 2:**
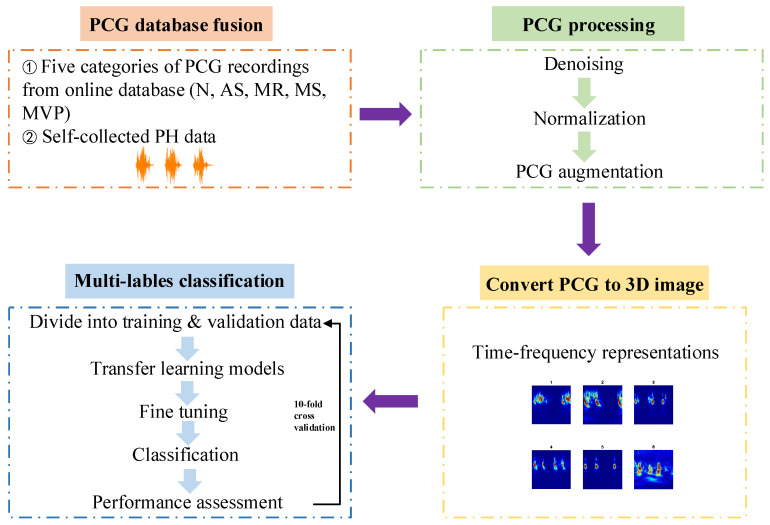
Flowchart of PCG classification by transfer learning models.

The main objective of this work is to apply transfer leaning networks to detect major cardiac diseases using HS recordings automatically. Figure 2 is the framework of this paper proposed approach. In summary, the research is divided into four steps: (1) Acquire the heart sound recordings, one is from the online database and the other one is PH subjects’ recordings collected by ourselves from the hospital; (2) Signal pre-processing including denoising, amplitude normalization and data augmentation; (3) One-dimensional heart sound signal is converted to three-dimensional time–frequency image which can help to improve the performance of the multi-classification results; (4) Apply transfer learning architectures to classify these images for training and testing the models in 10-fold cross validation. The proposed flow path could be used for multi-classification diagnosis of major heart diseases by PCG signals automatically.

The details on the programming used in the experiments are already uploaded to GitHub. Please check the website: https://github.com/wangmiao1992/pulmonary-hypertension-database/tree/main (accessed on 12 January 2022).

Furthermore, the software platform to run the proposed method is based on Matlab 2021a.

### 3.1. Signal Preprocessing

The sampling frequency of database A is 8000 Hz. However, the sampling frequency of database B is 2000 Hz. We only conduct preprocessing of database B and retain the original signal of database A. To eliminate the difference in sampling frequency, the sampling frequency of database A is reduced to 2000 Hz. Then, each heart sound signal in the two databases has fixed sample length of 2312. 

The signal quality of the database A is good, while the heart sound recordings from database B include slight noise. As we know, the frequency of heart sound signal is usually between 50 Hz and 150 Hz [28]. Digital filters can be used to remove the low- and high-frequency components. In this paper, the HS signals pass a third-order Butterworth filter with bandwidth in the range of 15 Hz to 150 Hz and reverses the filtered sequence and runs it back through the filter to remove the noise outside the bandwidth and avoid time delay. Subsequently, the signals in both database A and database B are normalized using Equation (1).

(1) $$X_{\mathrm{norm}}=\frac{x}{\left|x_{\max}\right|}$$

### 3.2. PCG Augmentations

Heart sound signal is a time series signal, its characteristics and individual differences hinder the application of the traditional data augmentation methods in the field of heart sound signal. Hence, how to explore a more effective and more suitable augmentation method from the original heart sound signal is an important problem for building of the multi-label heart sound diagnosis system.

The operation process of data augmentation usually included flip, rotate/reflection, shift, zoom, contrast, color and noise disturbance [29,30,31]. However, these data augmentation methods in the image field only change basic information such as position and angle from a macro perspective, and these methods can only apply in the field of simple computer vision methods such as image recognition, which can not be applied to data augmentation of heart sound signals.

In this research, the PCG augmentation method applies a 1D signal augmentation mechanism. The augmentation method includes HS signals under various cases in order to recognize the model with stronger generalization performance. The methodology explores background formations, and at the same time the transfer learning models are able to categorize various heart sound signals even in a noisy circumstance.

**Figure 3:**
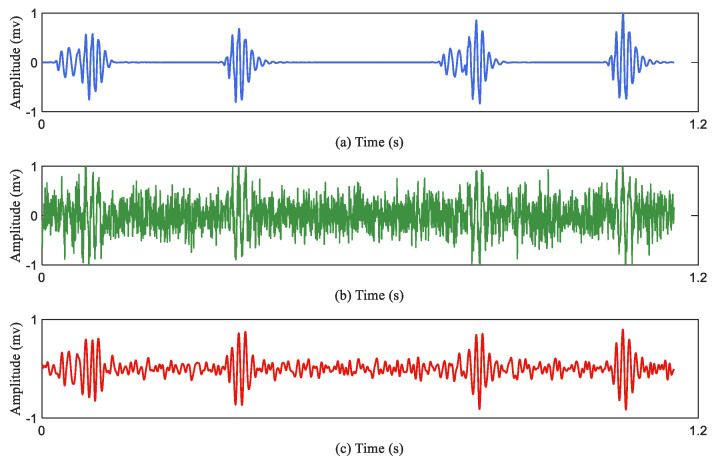
Examples of original PCG, augmented PCG and denoised PCG. (**a**) An original PCG signal (normal heart sound signal) after denoising; (**b**) an augmented PCG signal with noise; (**c**) a denoised PCG signal.

There is a given heart sound signal represented as ‘original_signal’. At the same time, the same-size background transformations are generated stochastically. The background transformation is displayed as ‘random_signal’, where the ‘delta’ represents the parameter of the deformation control. The background deformation ‘delta’ belongs to the interval “(0,1)". An augmented signal is calculated based on Equation (2), which is generated based on the random background noise mixed with the original heart sound signal. It should be noted that in the testing unit there is no data augmentation. Figure 3 describes the effect of the data augmentation. Figure 3a represents the original heart sound signal; Figure 3b represents the augmented heart sound signal through the Equation (2); Figure 3c represents the denoised signal of the Figure 3b. Table 3 summaries the recording distribution after data augmentation. Finally, the database contains 2400 PCG recordings in total. There are 400 PCG recordings in each class.

(2) augmentation_signal=original_signal+delta∗random_signal 

### 3.3. Creating Time–Frequency Representations

Time–frequency transformation is a common approach in the classification of speech events to extract a time–frequency representation of sound. Time–frequency representation is to convert a one-dimensional signal into a three-dimensional image representation. After that, the features extracted from the transformation are used to identify the most likely source of sound. Based on the investigation in [32], the authors conclude that among three time–frequency representations (short-time Fourier transform (STFT), Wigner distribution, and continuous wavelet transform (CWT)), CWT gives the clearest presentation of the time–frequency content for PCG signals.

The CWT spectrogram is produced by Morse analysis. A magnitude spectrogram of the heart sound signal is calculated for each sample. These spectrograms are used to train and test the transfer learning models. The CWT of a heart sound signal x(t) is defined in (3), and Equation (4) is the Morse analytic wavelet:

(3) $$W(a,b)=\int_{-\infty }^{\infty }x(t)\frac{1}{\sqrt{a}}\psi (\frac{t-b}{a})dt$$

(4) $$\psi (t)=e^{-t_{}^{2}}\cos(\pi \sqrt{\frac{2}{\ln2}}t)$$

where x(t) is a heart sound signal, ψ(t) is the mother wavelet, and a and b are the parameters that manage the scaling and translation of the wavelet, respectively. The CWT is calculated by varying a and b continuously over the range of scales and the length of the heart sound signal, respectively.

**Figure 4:**
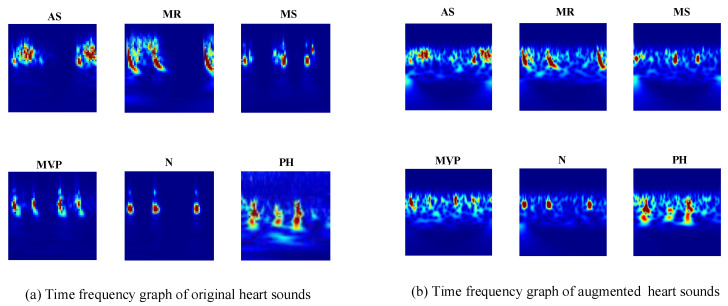
Time–frequency representation of PCG (**a**) time–frequency graphs of original heart sound signals; (**b**) time–frequency graphs of the augmented heart sound signals.

The CWT provide superior time and frequency resolution. This allows for different-sized analysis windows at different frequencies. The spectrograms of the heart sound signals show the frequencies at different times and provide an optical presentation that can be used to tell apart the various heart sounds. The CWT creates 3D scalogram data and they are stored as RGB images. To match the inputs of different transfer learning architectures, each RGB image is resized to an array of size n-by-m-by-3. For example, for the GoogLeNet architecture, the RGB image is resized to an array of size 224-by-224-by-3. The six typical spectrograms of HS signal are shown in Figure 4. Figure 4a represents the spectrogram of the original heart sound signals; Figure 4b represents the spectrogram of the augmented heart sound signals.

### 3.4. Architecture of Transfer Learning for PCG Multiple Classification

Transfer learning aims at utilizing the acquired knowledge on target domains to address other problems in different but related areas. This approach may be a better choice than some simple structures of CNN models. However, transfer learning is rarely reported and considered for classifying PCG signals.

**Figure 5:**
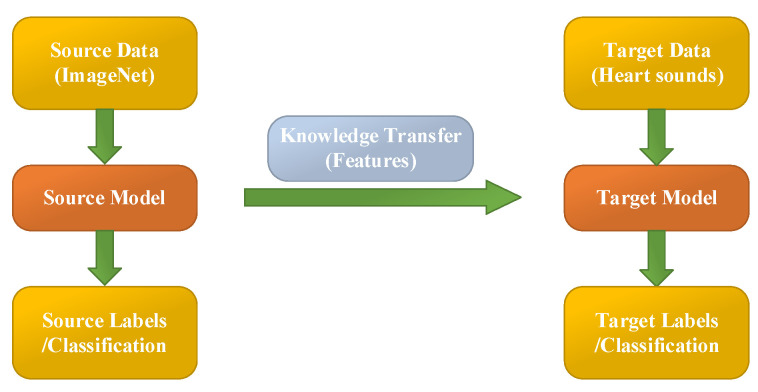
The process of transfer learning.

In this work, the heart sound signal is converted into its corresponding pattern based on CWT spectrogram, and two syncretic databases of HS signals are taken as one database to perform experimentation. The 10 existing transfer learning models (Squeezenet, Googlenet, NasNet-Large, Inceptionv3, Densenet201, DarkNet19, Mobilenetv2, Resnet101, Xception and Inceptionresnetv2) are used to classify the heart sound signals into six categories (N, AS, MR, MS, MVP, PH). These parameters of transfer learning models are shown in Table 4. It is worth noting that the different transfer learning models have different image input sizes, therefore the generated images should follow the input size of the models. Table 4 shows the image input sizes of the models. Additionally, Figure 5 illustrates the flow chart of transfer learning.

In this research, the pre-trained transfer learning networks’ parameters are modified and some of the architectures are fine-tuned. The earlier layers identify more common features of images, such as blobs, edges, and colors. Subsequent layers focus on more specific characteristics in order to differentiate categories.

For example, the original GoogLeNet is pretrained to categorize various pictures into 1000 target categories. However, in this research filed, we retrain GoogLeNet for solving the problem of PCG classification. To prevent over-fitting of the transfer learning model, a dropout layer is used. The final dropout layer (‘pool5-drop_7x7_s1’) is replaced for a dropout layer of probability 0.6. Furthermore, we also replace the layers of ‘loss3-classifier’ and ‘output’ with a new fully connected layer to adapt to the new data. At last, the learning rate factor is increased to 0.001. This is an iterative processing for training a neural network for minimizing the loss function. A gradient descent algorithm is used to minimize the loss function. In each reiteration, the loss function gradient is assessed, and at the same time the weight of the drop algorithm is updated. We set mini-batch size to 10 and max epochs to 15. In this paper, the stochastic gradient descent with momentum optimizer is applied. The other transfer learning models have the same settings as above.

The analysis and model development are performed in a workstation with hardware/software configuration and specification as follows: DELL(R) Precision T3240 i7-10700, Graphical Processing Units (GPU) NVIDIA Quadro RTX3000, 64GB RAM, and 64-bit Windows 10.

### 3.5. Model Training and Testing

The proposed methods used diverse HS data for training, validation and testing. The training and testing are based on 10-fold cross validation. In this research, nine folds are used for training the transfer learning models while one fold is used for testing. The process iterates repeatedly to ensure the coverage of the entire database for training and testing conditions. The choice of each fold is not based on independent subjects, because all the recordings of the patients are put in one collection. The training data includes all of the augmented data and 90% of the original heart sound data (There are 3800 heart sound recordings which include 2000 augmented heart sound recordings and 1800 original heart sound recordings). The testing data only include 10% of the original heart sound data (includes 200 original recordings).

### 3.6. Assessment Indicators

To evaluate the performance of the methodology in this paper, four indicators, accuracy, precision, recall and Fl-score, are used. Accuracy (ACC) is the indicator of all the correct recognition events. Precision and recall are powerful estimations when the database is quite imbalanced. Additionally, the F1_score is defined as the harmonic mean of precision and recall. The equations of the performance are calculated as follows:

(5) $$ACC=\frac{True\_Positive+True\_negative}{True\_Positive+True\_negative+False\_Positive+False\_negative}$$

(6) $$Precision=\frac{True\_Positive}{True\_Positive+False\_Positive}$$

(7) $$Recall=\frac{True\_Positive}{True\_Positive+False\_negative}$$

(8) $$F1\_score=2\times \frac{Precision\times Recall}{Precision+Recall}$$

## 4. Experiment Results and Discussions

### 4.1. Experiment Results

Table 5 shows one transfer learning model’s result—the accuracy with loss of the GoogleNet training and testing in the augmented PCG database and the original PCG database, respectively. The experiment is performed on the 10-fold cross validation. Here, in Table 5, there are some parameters, such as train samples, test samples, training accuracy (Acc), testing accuracy (Val Acc), training loss (Loss) and testing loss (Val Loss) on each fold. Table 5a shows 10-fold cross validation results on the augmented PCG database. Table 5b shows 10-fold cross validation results on the original database. It is visible from the Table 5a,b that the proposed methods achieve an average of 98% accuracy in classification of six categories both with the augmented data training and the original data training. The results of the PCG database show the efficiency of this method. Furthermore, we also evaluate the impact of the PCG augmentation method with additional background deformation.

**Figure 6:**
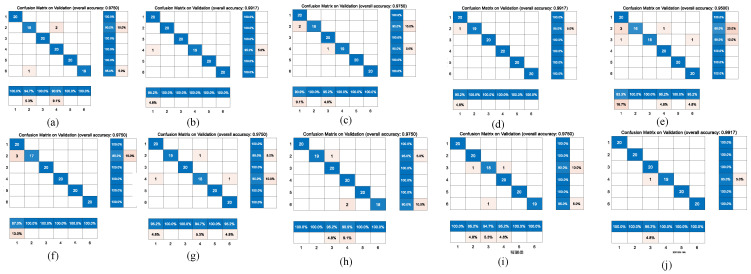
10-fold cross validation of confusion matrix for the GoogleNet model on augmentation database. The accuracy of each fold is: (**a**): 0.98; (**b**): 0.99; (**c**): 0.98; (**d**): 0.99; (**e**): 0.95; (**f**): 0.98; (**g**): 0.98; (**h**): 0.98; (**i**): 0.98; (**j**): 0.99.

**Figure 7:**
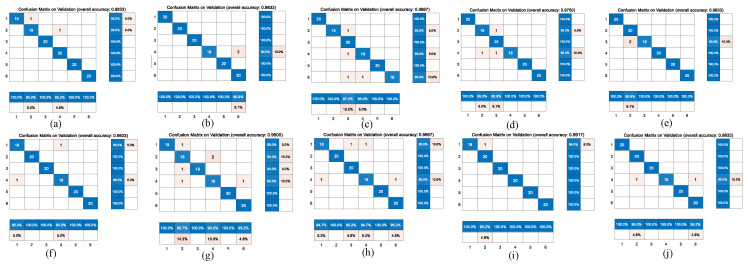
10-fold cross validation of confusion matrix for the GoogleNet model on original heart sound database. The accuracy of each fold is: (**a**): 0.98; (**b**): 0.98; (**c**): 0.97; (**d**): 0.98; (**e**): 0.98; (**f**): 0.98; (**g**): 0.95; (**h**): 0.97; (**i**): 0.99; (**j**): 0.98.

Figure 6 and Figure 7 represent the confusion matrix for the entire 10-fold with multiple classification estimations. At the same time, Table 6 shows the performance (precision, recall and Fl-score) of the GoogleNet structure for the multiple classifications of different heart diseases for all 10 folds with various indicators on the augmented PCG database and on the original PCG database.

**Figure 8:**
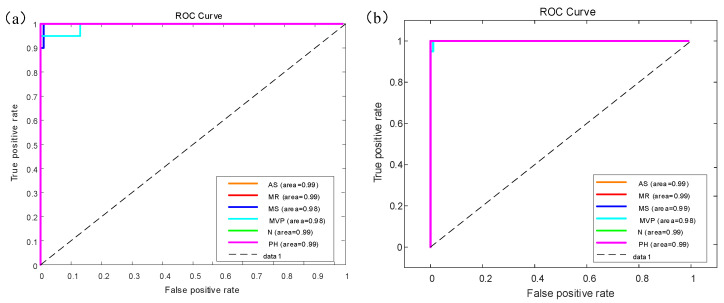
ROC curve for (**a**) the augmented heart sound classification based on GoogleNet; (**b**) the original heart sound classification based on GoogleNet.

Figure 8 shows the receiver operating characteristic (ROC) curve for the GoogleNet model results of multiple classifiers for six categories of heart sounds with AUC area. There are six colors which represent different categories of heart sounds. Figure 8a shows the ROC curve on the augmented PCG database; Figure 8b shows the ROC curve on the original PCG database.

**Figure 9:**
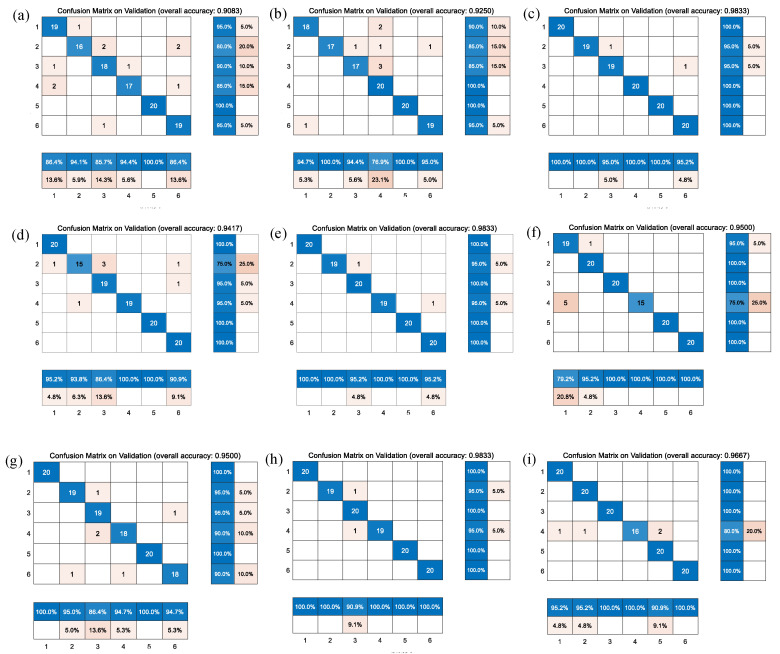
Comparison of confusion matrix results of nine categories of transfer learning models. (**a**) Xception convolutional neural network; (**b**) NASNet-Large convolutional neural network; (**c**) resnet101; (**d**) inceptionv3; (**e**) densenet201; (**f**) Inception-ResNet-v2; (**g**) mobilenetv2; (**h**) darknet; (**i**) squeezenet.

Figure 9 shows the comparison of the different confusion matrices for the multi-classification by the other transfer learning models, such as Xception convolutional neural network, NASNet-Large convolutional neural network, resnet101, inceptionv3, densenet201, Inception-ResNet-v2, mobilenetv2, darknet, and squeezenet. Table 7 presents the accuracy, recall, precision, and F1-scores corresponding to these transfer learning models, where the results show that the Resnet101, Densenet201, Darknet and the model before we used GoogleNet obtained good accuracy in comparison with peer approaches.

### 4.2. Experiment Discussions

The methods proposed by the authors have only considered one depiction of heart sound signals, which is spectrogram from the HSs. The spectrogram is an image representation of sound signals in the time–frequency domain. The inputted heart sound signals have been first converted into the respective spectrogram and then are classified further into six categories using the transfer learning models.

In addition, Table 8 describes the other methods without transfer learning. It shows the accuracy of other methods compared with the transfer learning models in the multiple classification of heart diseases. The comparison of these different methods is performed on the same database. However, the result from Table 8 shows that the accuracy is very low. We conduct two controlled trials: (1) Original 1D PCG signals are inputted into the Bi-LSTM network for six categories of heart sound classification, but an accuracy of only 21.67% is achieved; (2) The 3D images of the heart sound spectrogram are inputted into the simple CNN network only with three convolution layers, and the accuracy is only 76.67%.

Compared with B-mode ultrasonography, nuclear magnetic resonance imaging, computed tomography and so on, phonocardiography has the characteristics of being non-invasive, non-destructive, good repeatability, simple operation and low cost, which could be applied for the prevention, preliminary diagnosis and long-term monitoring of related diseases. With the development of digital medical technology and biological technology, researchers have increased the demands on the processing and analysis of heart sound signal in related fields. Automatic analysis methods for processing of medical sequence signals can share the responsibility and pressure of the medical domain, and provide long-term monitoring of disease. At the same time, they can help medical staff to grasp the condition better then work out plans for disease prevention and treatment. Thereby, doctors can enhance the overall health of society.

Despite advancements in the automatic diagnosis of heart sounds domain, there are still some limitations to be overcome to develop this technology further. For example, database deficiencies, huge feature extraction and low accuracy in multiple classification of heart disease. Solving these challenges can allow deep-learning technology to obtain a huge breakthrough in the field of human health. In our paper, we provide a heart sound database of pulmonary hypertension which is the first heart sound database related to pulmonary hypertension. Furthermore, feature extraction of heart sounds often takes a lot of time to acquire, which is a limitation. For this reason, we also proposed one-dimensional signal transfer to the three-dimensional image for training and testing, which can generate features automatically by a convolution layer in the heart sound domain. At last, we propose transfer learning technologies to diagnose multiple heart sounds and obtain a good performance. This overcomes the independent learning pattern through applying previously learned knowledge to solve similar problems. It is important for small-sample-size data to use transfer learning in the artificial intelligence domain because the pre-trained weights can be more efficient in training and obtain a better performance.

In this work, we suspect that the diversity of the augmented data can contribute to the networks’ ability to generalize to unseen data during the training stage. Data augmentation can improve the robustness of training. Comparison is made with traditional methods and transfer learning. In all experiments, the transfer leaning networks performed better on the task than other simple networks (such as Convolutional Neural Network and Long Short-Term Memory Network), as shown by several performance metrics. This approach has the potential to provide physicians with an efficient and accurate means to triage patients.

The proposed approach would have a significant impact in clinical situations by assisting medical doctors in decision making regarding different kinds of heart diseases. Our model performs efficiently in predicting the occurrence of an abnormality in a recorded signal. Moreover, it is tested on specific valvular diseases and patients with pulmonary hypertension who are not easily diagnosed early.

In summary, there are three contributions of this work. (1) The first is that we provide a new type of heart sound database (PH database). Additionally, our methods are validated under different conditions of HS databases. (2) The second is that we use a HS data augmentation strategy for completely automatic heart disease diagnosis. The method of augmentation improves the robustness of the heart diseases diagnosis, especially in noisy environments. (3) According to the published literature, transfer learning is rarely applied in the field of heart sound classification. We use 10 transfer learning models to verify the classification methods. We obtain a low error rate and great accuracy (0.98 accuracy for six categories of heart sounds) for multiple classification of heart diseases, which help to cope with multiple classification issues. 

## 5. Conclusion

Heart sound signals carry important information about the function of heart valves during heartbeats. Therefore, these signals are very important in diagnosing heart problems at an early stage. To detect heart problems with great precision, we apply the transfer learning architectures based on the CWT method under background deformation for classifying PCG. In total, we use 10 transfer learning models to build the methods. The classification results are good even they are validated by a fusion of two different databases. The results show the method is robust. This may be particularly useful in remote areas or community hospital screening activities.

## Figures and Tables

**Table 1:** A comparative performance of existing work for the cardiac disease classification.

Year	Related Work	Database	Condition	Feature Extraction	Method	Accuracy
2021	Haoran Kui et al. [17]	Collected by themselves	Two and four classes	MFSC	CNN	93.89% (two-classes); 86.25%(multi-classes)
2021	Omer Deperlioglu et al. [18]	PASCAL B-training	Three classes	Instant energy	Stacked autoencoder network	99.61%
2020	Neeraj Baghel et al. [21]	Yaseen database	Five classes	7-conv-layers	Improved network architecture	98.60%
2018	Yaseen et al. [22]	Yaseen database	Five classes	MFCC+DWT	SVM, DNN, KNN	97%
2021	Suyi Li et al. [23]	PhysioNet database	Two classes	Time–frequency feature fusion	Lightweight neural network model	95.50%
2021	Vinay Arora et al. [24]	PhysioNet 2016 and PASCAL 2011	Two classes	CWT	MobileNet, Xception, VGG16, ResNet, DenseNet, and InceptionV3	92.96%
2021	Turker Tuncer et al. [25]	Yaseen database	Five classes	Petersen graph pattern	Decision tree, linear discriminant, bagged tree, and support vector	100%
2021	Mohanad Alkhodari et al. [26]	Yaseen database	Five classes	Maximal overlap discrete wavelet transform	CNN-BiLSTM network	97.87%
2018	Omer Deperlioglu et al. [27]	PASCAL	Three classes	Heartbeat	CNN	97.9%

Note: MFCC (Mel-Frequency Cepstrum Coefficient); MFSC (Mel-frequency spectral coefficients); DWT (Discrete Wavelet Transform).

**Table 2:** Original heart sound database.

Heart Disease	Recording Size	Sample Frequency
Normal (N)	200	8000 Hz
Aortic Stenosis (AS)	200	8000 Hz
Mitral Regurgitation (MR)	200	8000 Hz
Mitral Stenosis (MS)	200	8000 Hz
Mitral Valve Prolapse (MVP)	200	8000 Hz
Pulmonary Hypertension (PH)	200 (with 74 subjects)	2000 Hz

**Table 3:** Recording distribution after data augmentation.

Heart Disease	Recording Size	Sample Frequency
Normal (N)	400	8000 Hz
Aortic Stenosis (AS)	400	8000 Hz
Mitral Regurgitation (MR)	400	8000 Hz
Mitral Stenosis (MS)	400	8000 Hz
Mitral Valve Prolapse (MVP)	400	8000 Hz
Pulmonary Hypertension (PH)	400	2000 Hz

**Table 4:** Overall view of the transfer learning models used in this study.

No.	Network	Depth	Size	Parameters (million)	Image Input Size
1	Squeezenet	18	5.2 MB	1.24	227 × 227 × 3
2	Googlenet	22	27 MB	7.0	224 × 224 × 3
3	Inceptionv3	48	89 MB	23.9	299 × 299 × 3
4	Densenet201	201	77 MB	20.0	224 × 224 × 3
5	Mobilenetv2	53	13 MB	3.5	224 × 224 × 3
6	Resnet101	101	167 MB	44.6	224 × 224 × 3
7	Xception	71	85 MB	22.9	299 × 299 × 3
8	Inceptionresnetv2	164	209 MB	55.9	299 × 299 × 3
9	nasnetlarge	*	332 MB	88.9	331 × 331 × 3
10	darknet19	19	78 MB	20.8	256 × 256 × 3

* The nasnetlarge networks do not consist of a linear sequence of modules.

**Table 5:** (**a**) Results of 10-fold cross validation on augmented heart sound database on GoogleNet. (**b**) 10-fold cross validation result on the original dataset on GoogLeNet.

**(a)**						
**Fold**	**Train Samples**	**Test Samples**	**Acc**	**Val Acc**	**Loss**	**Val Loss**
1	2280	120	1.00	0.99	0.10	0.07
2	2280	120	1.00	0.99	0.12	0.10
3	2280	120	1.00	0.98	0.09	0.02
4	2280	120	1.00	0.95	0.14	0.12
5	2280	120	1.00	0.98	0.19	0.04
6	2280	120	1.00	0.98	0.15	0.04
7	2280	120	1.00	0.98	0.13	0.06
8	2280	120	1.00	0.98	0.16	0.14
9	2280	120	1.00	0.98	0.16	0.05
10	2280	120	1.00	0.99	0.10	0.07
Mean				0.98		
**(b)**						
**Fold**	**Train Samples**	**Test Samples**	**Acc**	**Val Acc**	**Loss**	**Val Loss**
1	1080	120	0.98	0.98	0.00	0.2
2	1080	120	1.00	0.98	0.00	0.3
3	1080	120	1.00	0.98	0.1	0.07
4	1080	120	1.00	0.95	0.00	0.02
5	1080	120	1.00	0.97	0.00	0.05
6	1080	120	1.00	0.97	0.00	0.08
7	1080	120	1.00	0.98	0.00	0.09
8	1080	120	1.00	0.99	0.00	0.10
9	1080	120	1.00	0.98	0.00	0.06
10	1080	120	1.00	0.98	0.00	0.06
Mean				0.98		

**Table 6:** Results for multiple class classification on GoogleNet.

Performance Indicators		Results on an Augmented Database						Results on an Original Database					
		AS	MR	MS	MVP	N	PH	AS	MR	MS	MVP	N	PH
Fold 1	Precision	0.95	1.00	1.00	1.00	1.00	1.00	1.00	0.95	1.00	0.95	1.00	1.00
	Recall	1.00	1.00	1.00	0.95	1.00	1.00	0.95	0.95	1.00	1.00	1.00	1.00
	F1-Score	0.98	1.00	1.00	0.97	1.00	1.00	0.97	0.95	1.00	0.98	1.00	1.00
Fold 2	Precision	0.95	1.00	1.00	1.00	1.00	1.00	0.95	1.00	1.00	0.95	1.00	1.00
	Recall	1.00	0.95	1.00	1.00	1.00	1.00	0.95	1.00	1.00	0.95	1.00	1.00
	F1-Score	0.98	0.97	1.00	1.00	1.00	1.00	0.95	1.00	1.00	0.95	1.00	1.00
Fold 3	Precision	1.00	0.95	0.95	0.95	1.00	1.00	1.00	1.00	1.00	1.00	1.00	0.91
	Recall	1.00	1.00	0.90	1.00	1.00	0.95	1.00	1.00	1.00	0.90	1.00	1.00
	F1-Score	1.00	0.98	0.92	0.98	1.00	0.97	1.00	1.00	1.00	0.95	1.00	0.95
Fold 4	Precision	0.83	1.00	1.00	0.95	1.00	0.95	1.00	0.86	1.00	0.90	1.00	0.95
	Recall	1.00	0.80	0.90	1.00	1.00	1.00	0.95	0.90	0.95	0.90	1.00	1.00
	F1-Score	0.91	0.89	0.95	0.98	1.00	0.98	0.97	0.88	0.97	0.90	1.00	0.98
Fold 5	Precision	1.00	1.00	0.95	0.91	1.00	1.00	1.00	1.00	0.87	0.95	1.00	1.00
	Recall	1.00	0.95	1.00	1.00	1.00	0.90	1.00	0.95	1.00	0.95	1.00	0.90
	F1-Score	1.00	0.97	0.98	0.95	1.00	0.95	1.00	0.97	0.93	0.95	1.00	0.95
Fold 6	Precision	0.91	1.00	0.95	1.00	1.00	1.00	0.95	1.00	0.95	0.95	1.00	0.95
	Recall	1.00	0.90	1.00	0.95	1.00	1.00	0.90	1.00	1.00	0.90	1.00	1.00
	F1-Score	0.95	0.95	0.98	0.97	1.00	1.00	0.92	1.00	0.98	0.92	1.00	0.98
Fold 7	Precision	0.95	1.00	1.00	0.95	1.00	0.95	1.00	0.95	0.91	1.00	1.00	1.00
	Recall	1.00	0.95	1.00	0.90	1.00	1.00	1.00	0.95	1.00	0.90	1.00	1.00
	F1-Score	0.98	0.97	1.00	0.92	1.00	0.98	1.00	0.95	0.95	0.95	1.00	1.00
Fold 8	Precision	0.87	1.00	1.00	1.00	1.00	1.00	1.00	0.95	1.00	1.00	1.00	1.00
	Recall	1.00	0.85	1.00	1.00	1.00	1.00	0.95	1.00	1.00	1.00	1.00	1.00
	F1-Score	0.93	0.92	1.00	1.00	1.00	1.00	0.97	0.98	1.00	1.00	1.00	1.00
Fold 9	Precision	1.00	0.95	1.00	0.91	1.00	1.00	1.00	0.91	1.00	1.00	1.00	1.00
	Recall	1.00	0.90	1.00	1.00	1.00	0.95	1.00	1.00	0.90	1.00	1.00	1.00
	F1-Score	1.00	0.92	1.00	0.95	1.00	0.97	1.00	0.95	0.95	1.00	1.00	1.00
Fold 10	Precision	1.00	1.00	0.95	1.00	1.00	1.00	1.00	0.95	1.00	1.00	1.00	0.95
	Recall	1.00	1.00	1.00	0.95	1.00	1.00	1.00	1.00	1.00	0.90	1.00	1.00
	F1-Score	1.00	1.00	0.98	0.97	1.00	1.00	1.00	0.98	1.00	0.95	1.00	0.98

**Table 7:** Nine transfer learning models of classification performance based on augmented heart sound database.

Models	ACC	Performance	Results on an Augmented Database					
			AS	MR	MS	MVP	N	PH
xception	0.90	Precision	0.86	0.94	0.86	0.94	1.00	0.86
		Recall	0.95	0.8	0.9	0.85	1	0.95
		F1-Score	0.90	0.86	0.88	0.89	1.00	0.90
Resnet101	0.98	Precision	1.00	1.00	0.95	1.00	1.00	0.95
		Recall	1.00	0.95	0.95	1.00	1.00	1.00
		F1-Score	1.00	0.97	0.95	1.00	1.00	0.98
NASNet_large	0.92	Precision	0.95	1.00	0.94	0.77	1.00	0.95
		Recall	0.90	0.85	0.85	1.00	1.00	0.95
		F1-Score	0.92	0.92	0.89	0.87	1.00	0.95
Inception-v3	0.94	Precision	0.95	0.94	0.86	1.00	1.00	0.91
		Recall	1.00	0.75	0.95	0.95	1.00	1.00
		F1-Score	0.98	0.83	0.90	0.97	1.00	0.95
DenseNet-201	0.98	Precision	1.00	1.00	0.95	1.00	1.00	0.95
		Recall	1.00	0.95	1.00	0.95	1.00	1.00
		F1-Score	1.00	0.97	0.98	0.97	1.00	0.98
Inception-ResNet-v2	0.95	Precision	0.79	0.95	1.00	1.00	1.00	1.00
		Recall	0.95	1.00	1.00	0.75	1.00	1.00
		F1-Score	0.86	0.98	1.00	0.86	1.00	1.00
MobileNet-v2	0.95	Precision	1.00	0.95	0.86	0.95	1.00	0.95
		Recall	1.00	0.95	0.95	0.90	1.00	0.90
		F1-Score	1.00	0.95	0.90	0.92	1.00	0.92
Darknet19	0.98	Precision	1.00	1.00	0.91	1.00	1.00	1.00
		Recall	1.00	0.95	1.00	0.95	1.00	1.00
		F1-Score	1.00	0.97	0.95	0.97	1.00	1.00
Squeezenet	0.97	Precision	0.95	0.95	1.00	1.00	0.91	1.00
		Recall	1.00	1.00	1.00	0.80	1.00	1.00
		F1-Score	0.98	0.98	1.00	0.89	0.95	1.00

**Table 8:** Results for multiple class classifications based on other deep-learning networks.

Models	Network Architecture	Features	Accuracy
Bi-LSTM	fully connected layer of size 2, a softmax layer, the maximum number of epochs is 30.	Original one-dimensional PCG signals	21.67%
CNN	3 convolution layer, each layer contains a normalized RELU, MaxPooling, fully connected layer, and Softmax. The maximum number of epochs is 15.	Spectrogram of the PCG signals	76.67%

## Data Availability

The data presented in this study are openly available in https://github.com/wangmiao1992/pulmonary-hypertension-database/tree/main (accessed on 12 January 2022).
